# Shaping ability of protaper next compared with waveone in late-model three-dimensional printed teeth

**DOI:** 10.1186/s12903-018-0573-8

**Published:** 2018-06-25

**Authors:** Zhi Cui, Zhao Wei, Minquan Du, Ping Yan, Han Jiang

**Affiliations:** 1 The State Key Laboratory Breeding Base of Basic Science of Stomatology (Hubei-MOST) & Key Laboratory of Oral Biomedicine Ministry of Education, School & Hospital of Stomatology, Wuhan University, Luoyu Road, Wuhan City, 237 China; 2 Department of Dentistry, Second Hospital of Baoding, 338 Dongfeng West Road, Baoding, China; 3 Department of Prevention Dentistry, School and Hospital of Stomatology, Wuhan University, 237 Luoyu Road, Wuhan, China

**Keywords:** Printed resin teeth, Three-dimensional S-shaped root canal, ProTaper next, WaveOne, Micro-computed tomography imaging

## Abstract

**Background:**

Comparison of the shaping ability of advanced nickel-titanium (Ni-Ti) instruments is of great interest to the field of endodontics. However, the models used to study canal preparation still lack uniformity, relevance to reality and complexity. The aim of this study was thus to compare the shaping abilities of the ProTaper Next (PN) and WaveOne (WO) Ni-Ti instruments in three-dimensional (3D)-printed teeth, which may overcome the present defects of most real teeth and model teeth including 3D S-shaped canals.

**Methods:**

Six teeth and their corresponding 3D-printed replicas were prepared using the same kind of Ni-Ti instrument. The pre- and post-preparation volumes, surface areas and transportation of the canals were measured to compare the teeth with their replicas. Twenty 3D-printed teeth with S-shaped canals were used to support the preparation study. The S-shaped canals were then scanned to measure their volumes and surface areas. Next, the two kinds of instruments were used to prepare the 3D-printed canals (*n* = 10 per group). The volume and surface area of the canals, the transportation along the two curvatures and the percentage of unprepared surface area were measured. Micro-CT and VGstudio2.2 (VG2.2) software were used to perform scans and collect data throughout the research. The paired-samples T test and Kruskal-Wallis H test were used for statistical analysis.

**Results:**

There was no significant difference between the real canals and the printed ones post-preparation (*P* > .05). The printed S-shaped root canals had a unified shape, with a small standard deviation and range. The WO group had higher mean values for the volume and superficial area measurements compared with the PN group (*P* < .05). No differences in the untouched areas were found between the two systems (*P* > .05). PN caused less transportation at the apical curve than WO did (*P* < .05).

**Conclusions:**

In conclusion, 3D-printed teeth are suitable for the study of Ni-Ti rotary instruments. Furthermore, the PN rotary system caused less transportation at the apical curve than the WO system did in complicated root canal procedures.

## Background

Shaping and cleaning root canals are essential parts of endodontic chemo-mechanical disinfection. Many experiments have focused on comparing the shaping abilities of nickel-titanium (Ni-Ti) instruments in extracted teeth and simulated root canals [1]. However, use of the former teeth cannot ensure uniformity [2], and the latter lack relevance to reality as well as complex morphology [3].

Three-dimensional (3D) printing is a novel technology that can change manufacturing methods. Combined with stomatological approaches, various appliances have been 3D-printed in the dental field, such as drill guides for dental implants; physical models for prosthodontics, orthodontics and surgery; and craniomaxillofacial and orthopaedic implants [4]. In a recent study, 3D-printed teeth were also introduced into preparation research [5, 6].

The ProTaper Next (PN; Dentsply Maillefer, Ballaigues, Switzerland) and WaveOne (WO; Dentsply Maillefer) systems are based on innovative metallurgy in which manufacturers introduce M-Wire alloy to improve the fatigue life and flexibility of the files. ProTaper Next is the successor of ProTaper Universal and has superior torque and speed. The off-centred rectangular cross-section gives the file a snake-like “swaggering” movement [7], which can generate an enlarged space for debris removal. WO is a single-file system, and combined with the balanced force technique, the file can turn a shorter angular distance. This motion reduces the file stress and plastic deformation [8].

Although these Ni-Ti instruments are advanced and popular in clinical treatment, few studies have researched which file system is more suitable for the treatment of complex root canals. In the present study, the shaping abilities of PN and WO were compared in 3D-printed teeth which contained unified 3D S-shaped root canals. The null hypothesis was that there would be no difference between the two Ni-Ti instruments in terms of the analysed parameters.

## Methods

### Selection and characterization of teeth

Extracted permanent molars and maxillary premolars with mature apices and no previous root canal therapy were selected from a pool of extracted teeth. Radiographic images of each tooth were acquired in the buccolingual and mesiodistal orientations. Based on the results, three maxillary first premolars and three mandibular first molars, with two narrow root canals (30–40°), were selected for the analysis [9]. Micro-CT (Scanco Medical, Bassersdorf, Switzerland), which outputs images in TIFF format and has a resolution of 30 μm, was used to scan these teeth. VGstudio Max version 2.2 (VG2.2) software (Volume Graphics, Heidelberg, Germany) was then used to manage the TIFF images into a 3D construction and to output data in STL format.

### Resin tooth creation using 3D printing technology

The machine, ProJet 3500 HDMax (3D Systems, South Carolina, America), used for 3D printing has a precision of 16 μm and can be used with two materials [4]. The printer use UV-curable plastic, VisiJet M3 Crystal (3D Systems, South Carolina, America), and support material, VisiJet S300 (3D Systems, South Carolina, America), which allow for hands-free, melt-away removal without damaging the delicate structures. Every tooth was created in duplicate, such that there were six experimental pairs, including twelve pairs of root canals.

### Root canal instruments

Both of the real and duplicate tooth were instrumented using a new suite of PN with permanent rotation at 300 rpm and 3 Ncm. All instruments were operated with an X-Smart Plus endodontic motor (Dentsply Maillefer). A #10 K-file (Dentsply Maillefer) was used to dredge the root canals and measure the working length, which was defined as 1 mm shorter than the distance from the reference plane to the point where the file tip was visible under the visual field of a microscope (Leica M320 F12, Germany). The PN X1 (size 17, .04 taper) and, X2 (size 25, .06 taper) were selected as the appropriate files because of the size of the root canals. The preparation processes were as follows:

1. X1 was moved in the apical direction using a 33 mm mm in-and-out motion and with light apical pressure. Sufficient irrigation was conducted after three or four motions. This process was repeated until the working length was completed.
2. X2 was used to complete the work using the same method.

### Micro-CT scanning and VG2.2 analysis

The VG2.2 was used to calculate the volume and surface area of each root canal by measuring the region of interest. Before preparation, the printed teeth were scanned by micro-CT, and the surface area and volume were measured using VG2.2. The post-preparation parameters were then determined using the same method. VG2.2 could overlap pre- and post-preparation samples through the best-matching function and determine the geometric centres by measuring the points on the border of the sections automatically. The transportation value of the canals was defined as the distance between the approximate centres of the fixed sections of the pre- and post-preparation root canals [10]. A dipstick was then used to help to measure the transportation at four sections, respectively located 1 mm, 3 mm, 5 mm, and 9 mm from the apex.

### Comparing shaping abilities of Ni-Ti instruments

A mandibular first molar was chosen to conform to antecedent methods and standards. This tooth had a complex distal root canal that contained two curves with different dimensions and positions. Utilizing the model tooth, twenty resin teeth were printed and used to measure the volume and surface area. The replicas were then separated into two groups (*n* = 10). The resin teeth in the PN group were prepared as previously described. The WO group was instrumented using a reciprocating working motion of 170° counterclockwise and 50° clockwise with a primary file (size 25, 0.08 taper). All the preparation operations were completed by a sole endodontist who was experienced in these techniques and who had been sufficiently trained using both true and printed teeth. Every resin tooth had its own suit of instruments. The preparation process for the WO system was as follows:

1. A single file was used to approximately shape the coronal two-thirds of the canal length with a progressive up-and-down movement with no more than three or four repetitions.
2. The canal was irrigated with copious distilled water.
3. The file was applied to the whole length using the same process. During the process, only a small amount of force was required.

The transportation and the proportion of the unshaped surface area were detected in the two curves of the S-shaped canal (the cervical half and the apical half). The un-instrumented surface area was measured by counting the number of static voxels in the non-overlapping area [11]. All of the results were obtained using VG2.2.

### Statistical analysis

The statistical analysis was performed with SPSS (IBM SPSS Statistics 21; SPSS Inc., Chicago, IL). The different parameters of the twelve pairs of root canals were analysed using the paired-samples T test or the Wilcoxon signed-rank test. The uniformity of the twenty printed canals was then assessed based on the standard deviation, maximum and minimum. Other multiple comparisons of the shaping abilities of the two instrument groups were analysed using the independent-samples T test or the Mann-Whitney U test. The statistical significance level was set at *P* < .05.

## Results

### Comparison of real root canals and 3D-printed canals

**Fig. 1:**
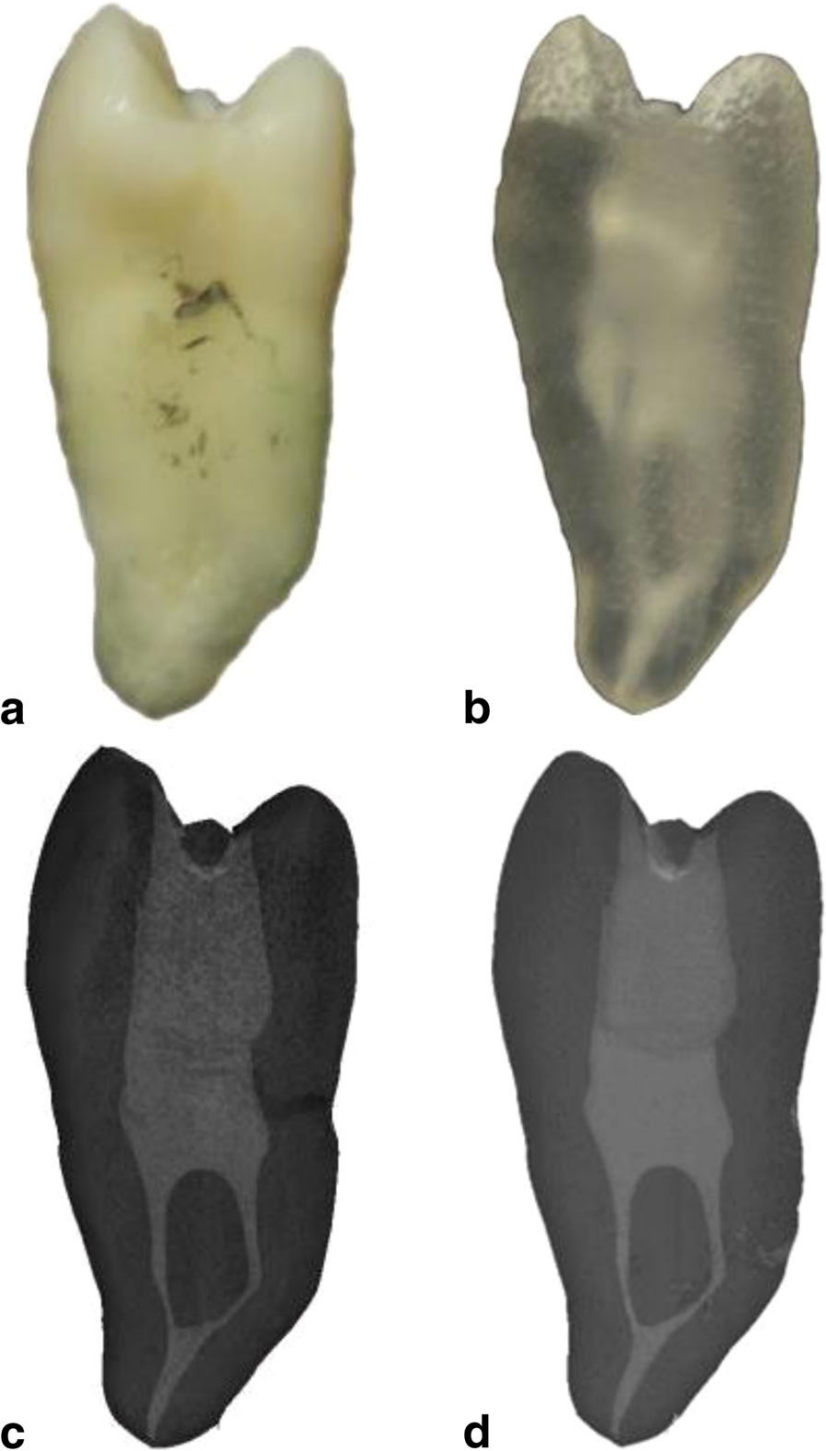
Images of a real premolar and its 3D-printed replica**. a** Image of the chosen premolar, **b** image of the 3D-printed tooth, **c** and **d** micro-CT images of (a) and (b), respectively

**Fig. 2:**
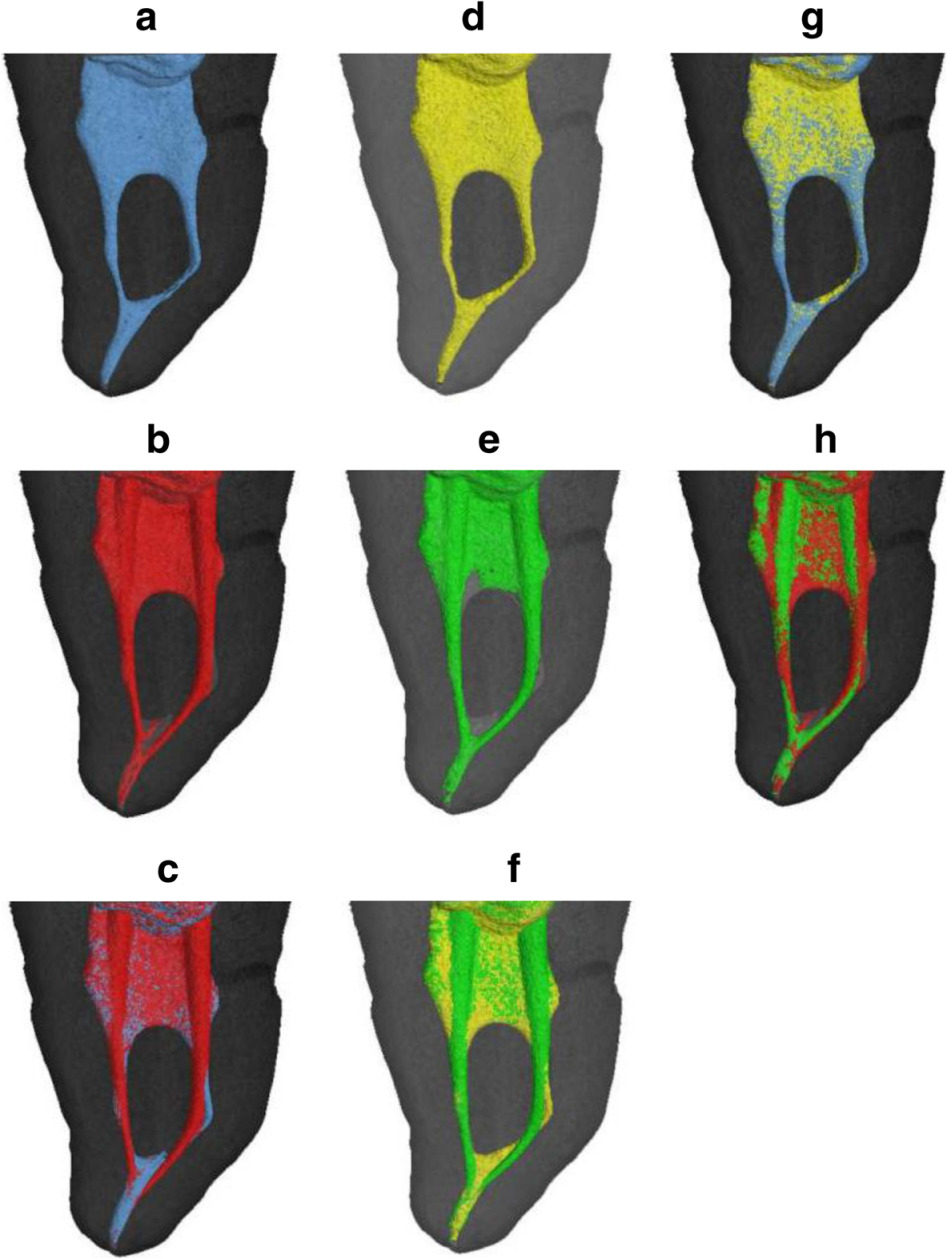
Images comparing the original premolar to a 3D-printed replica tooth. (**a**) (**b**) (**c**) Images of the root canal of a premolar, showing the pre-preparation canals in blue and the post-preparation areas in red. (**c**) The untouched areas are presented after overlapping (**a**) and (**b**). Pre-preparation, post-preparation and overlapping root canal images for the 3D-printed tooth are shown in yellow (**d**), green (**e**), and (**f**), respectively. (**g**) and (**h**) show overlapping canal images for the real premolar and the 3D-printed tooth before and after preparation, respectively

Figure 1 shows one pair of the six pairs of real teeth and their 3D-printed counterparts. Before preparation, the volume and surface area of the real canals were not significantly different from those of the printed canals (*P* > .05) (Table 1). In addition, similarly, after preparation, the volumes and surface areas of the true canals and duplicates were not significantly different (*P* > .05) (Table 1). There was no significant difference in the transportation of the real root canals and the duplicate canals in any of the four sections (*P* > .05) (Table 2). Overlapping images of the root canals of the chosen pair of teeth are shown in Fig. 2.

**Table 1:** Volumes and surface areas of real and 3D-printed canals pre- and post-preparation (*n* = 12)

Group	Volume before preparation (mm^3^)	Volume after preparation (mm^3^)	Surface area before preparation (mm^2^)	Surface area after preparation (mm^2^)
	Mean ± SD	Mean ± SD	Mean ± SD	Mean ± SD
Real teeth	2.82 ± .74	2.94 ± .75	19.61 ± 3.65	20.51 ± 3.58
3D-printed teeth	2.78 ± .73	2.97 ± .77	19.57 ± 3.60	20.59 ± 3.24
*P* value	.289^b^	.064^a^	.387^b^	.494^a^

^a^Paired-samples T test (*P* < .05)

^b^Wilcoxon signed-rank test (*P* < .05)

**Table 2:** Transportation in four sections of real and printed canals after preparation (*n* = 12)

Group	1 mm from apex (mm)	3 mm from apex (mm)	5 mm from apex (mm)	9 mm from apex (mm)
	Mean ± SD	Mean ± SD	Mean ± SD	Mean ± SD
Real teeth	.047 ± .037	.053 ± .042	.052 ± .052	.013 ± .014
3D-printed teeth	.053 ± .042	.06 ± .053	.056 ± .056	.014 ± .016
*P* value	.084^b^	.058^b^		.655^b^

^b^Wilcoxon signed-rank test (*P* < .05)

### Twenty 3D-printed root canals

**Fig. 3:**
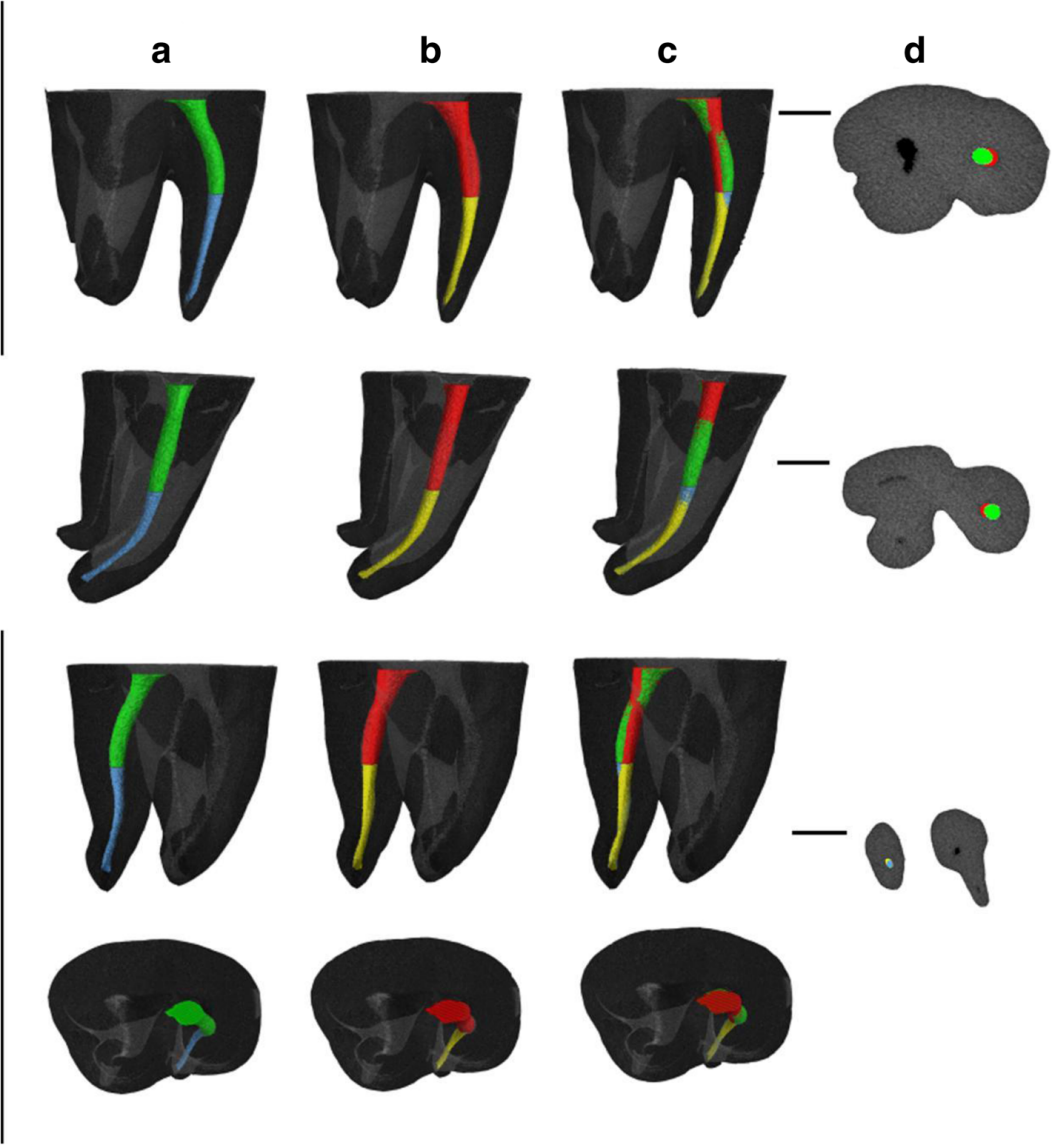
The different directions of the overlapping images of the S-shaped root canals. The coronal curvatures of the pre- and post-preparation canals are shown in green and red, respectively. The pre- and post-preparation apical regions are shown in blue and yellow, respectively. Column **a** shows images of the prepared root canals, and images of the post-preparation canals are shown in column **b**. In column **c**, the untouched areas are presented after overlapping the images in the **a** and **b** columns. Finally, column **d** shows the sections of the overlapping canals

Different views of the construction of the S-shaped root canal can be seen in Fig. 3. The mean volume of the original simulated root canals was 1.92 ± .03 mm^3^, with a minimum value of 1.87 mm^3^ and a maximum value of 1.95 mm^3^. The mean surface area of the pre-prepared root canals was 19.00 ± .13 mm^2^. Here, 19.26 mm^2^ and 18.81 mm^2^ were the minimum and maximum values, respectively.

### Volume and surface area in two groups

The volume and surface area values in the WO group were greater than the values in the PN group (*P* < .05) (Table 3).

**Table 3:** Volumes and surface areas of post-preparation simulated canals (*n* = 10)

Instrument	Volume (mm^3^)		Surface area (mm^2^)	
	Mean ± SD	Min-Max	Mean ± SD	Min-Max
ProTaper Next	3.21 ± .04	3.16–3.27	21.22 ± .77	20.24–22.70
WaveOne	3.37 ± .06	3.24–3.46	22.67 ± .61	21.89–23.63
*P* value	<.001^d^		<.001^c^	

^c^Independent-samples T test (*P* < .05)

^d^Mann-Whitney U test (*P* < .05)

### Percentage of unshaped area in two groups

Based on the data shown in Table 4, both groups had remaining unshaped areas in the apical and coronal halves. Notably, there was more untouched area in the coronal half compared with the apical half. However, for the untouched proportion of the two halves of the canals, there was no difference between the groups (*P* > .05).

**Table 4:** Percentages of unprepared areas in the apical and coronal halves of canals (*n* = 10)

Instrument	Unprepared apical area (%)		Unprepared coronal area (%)	
	Mean ± SD	Min-Max	Mean ± SD	Min-Max
ProTaper Next	11.03 ± .96	10.11–12.8	55.70 ± 1.25	53.26–57.19
WaveOne	12.06 ± 1.21	9.25–13.10	55.53 ± 1.01	53.48–57.24
*P* value	.683^c^		.684^c^	

^c^Independent-samples T test (*P* < .05)

### Transportation in two groups

The mean transportation in the PN group at the apical curve was less than that at the cervical curve. A similar result was obtained in the WO group (Table 5). WO caused more transportation at the apical curve than PN did (*P* < .05); however, there was no difference between the two groups at the cervical curve (*P* > .05).

**Table 5:** Transportation at the apical and coronal curvatures (*n* = 10)

Instrument	Apical curvature (mm)		Coronal curvature (mm)	
	Mean ± SD	Min-Max	Mean ± SD	Min-Max
ProTaper Next	0.14 ± .03 0.19	0.09–0.20	0.41 ± .04 0.43	0.37–0.50
WaveOne	±.05	0.11–0.27	±.06	0.35–0.50
*P* value	.014^c^		.404^c^	

^c^Independent-samples T test (*P* < .05)

## Discussion

The use of 3D-printed teeth in this type of comparative study, which assessed the shaping abilities of two Ni-Ti instrument systems, is a novel application of this powerful technology. In previous studies, researchers used extracted teeth to more accurately simulate clinical conditions [7, 10, 12–18]. However, real teeth have unique root canal systems, interfering with the uniformity of experiments [19]. Collection of the appropriate human teeth for in vitro examination and measurement of the curvature parameters for classification also make the experiments much more complicated. Other studies have used resin blocks [4, 20–25], but the physical properties of resin include greater hardness than for dentin [26]. Additionally, the simulated constructions do not reflect the diversity of real root canal morphology [27]. The frequency of instrument failure and the duration of the procedure using such simulated teeth are also not generalizable to clinical situations [28].

In the present study, duplicate teeth were successfully 3D printed based on scanning and digital reconstruction. No differences were found in the post-preparation volume, surface area or transportation between the 3D-printed teeth and the real teeth. This finding suggests that 3D-printed teeth could be suitable for comparing the shaping abilities of different Ni-Ti instruments. One of the advantages of 3D-printed teeth is that they can provide the uniformity and relevance to reality. Thus, the data from this research may be applicable to real-life clinical operations. The volumes of the true canals and duplicates were not significantly different after the preparation, however, the *p*-value (*p* = 0.064) is close to the significativity. The posibility is that the material of printed tooth need more similar of physical properties and bio-performences with natural dentin.

Earlier studies on the morphology of root canals have indicated that nearly all canals have two curvatures [29]. Other studies have demonstrated that an S-shaped root canal increases the difficulty of preparation and the risk of instrument fracture [20]. Compared with previous experimental root canals with single [7, 12, 15–18, 23, 30] or two-dimensional S-shaped [20, 21, 23, 24, 26] curvatures, printed teeth with a stereo S-shaped root canal can simulate the clinical conditions and challenges of preparing such a canal. With the progress of 3D printing technology, our hope is that computer design technology will be able to adjust the degree, radius, location and other shape details of curvatures to satisfy different study criteria.

Utilizing 3D-printed teeth, the shaping abilities of two NiTi instruments were compared. In contrast to the results of earlier research with extracted teeth [12, 13], the WO primary file removed more dentin and yielded a larger post-preparation surface area than the PN X2 did. In previous studies, although the degree and radius of the original curvature of true teeth were considered when allocating the teeth into groups, the volume and surface area of the canals were not sufficient to differentiate the teeth. Differences in canal morphology may affect canal preparation, and positive results may be influenced by discrepancies between canal shapes [31, 32]. The two indexes thus cannot be measured in resin blocks using a two-dimensional method.

In the current study, both groups exhibited a greater unshaped area in the cervical curvature region than in the apical region. This result was similar to that of a study by Cabanilas using WO and other instruments [33]. The finding may be due to the fact that the root canals had a capacious oval part in the coronal half and a conspicuous constriction in the apical half. This consideration implies that the selection of the appropriate primary file should be based on the integral root shape of the tooth in question. The remaining smear layers require sufficient chemical irrigation [34].

Both instrument groups cause more transportation in the coronal curves and less in the apical ones. This finding is corroborated by the results of previous experiments [14, 15, 21], which revealed a decreased tendency towards transportation from the cervical part to the apical part. The reason for this result may be that a longer diameter for the file predisposes it to resist deformation forces and straighten the coronal part. We observed the same phenomenon as that described in the results of Zhao’s study [12]: the WO system produced more transportation at the apical curvature than the PN system did. The PN X1 (size 17, 0.04 taper) plays a key role in the preliminary enlargement of the apical part of the canal, which reduces the degree of curvature and the pressure on the main file [35]. For the WO system, the relatively larger taper of the primary file increases the degree of transportation [7, 36, 37]. However, Davut’s study [13] indicated that the two instrument systems produce similar levels of transportation in the apical half of curved canals. The various software programs and methods that are used for measurement affect the accuracy of studies, and the transportation data vary considerably between the available published studies. The widely accepted measurement method should therefore be further discussed and researched.

## Conclusions

Here, 3D-printed teeth were found to be suitable for the study of Ni-Ti instruments. Under the study conditions, the null hypothesis was rejected. PN had superior function in reducing transportation in the apical potion of the complex root canal system. Both instruments adhered to the original simulated root canal shape, however, unshaped areas still existed in the root canal systems.

## Abbreviations

3D: three-dimensional
Ni-Ti: Nickel-titanium
PN: Protaper next
VG2.2: VGstudio2.2
WO: Waveone
